# Covalent ISG15 conjugation positively regulates the ubiquitin E3 ligase activity of parkin

**DOI:** 10.1098/rsob.160193

**Published:** 2016-08-17

**Authors:** Eunju Im, Lang Yoo, Minju Hyun, Woo Hyun Shin, Kwang Chul Chung

**Affiliations:** Department of Systems Biology, College of Life Science and Biotechnology, Yonsei University, Seoul 03722, South Korea

**Keywords:** Parkinson's disease, parkin, ISG15, ISGylation, E3 ligase, HERC5

## Abstract

Parkinson's disease (PD) is characterized by selective loss of dopaminergic neurons in the pars compacta of the substantia nigra and accumulation of ubiquitinated proteins in aggregates called Lewy bodies. Several mutated genes have been found in familial PD patients, including *SNCA* (α-synuclein), *PARK2* (parkin), *PINK1*, *PARK7* (DJ-1), *LRRK2* and *ATP13A2*. Many pathogenic mutations of *PARK2*, which encodes the ubiquitin E3 ligase parkin, result in loss of function, leading to accumulation of parkin substrates and consequently contributing to dopaminergic cell death. ISG15 is a member of the ubiquitin-like modifier family and is induced by stimulation with type I interferons. Similar to ubiquitin and ubiquitination, covalent conjugation of ISG15 to target proteins (ISGylation) regulates their biochemical properties. In this study, we identified parkin as a novel target of ISGylation specifically mediated by the ISG15-E3 ligase HERC5. In addition, we identified two ISGylation sites, Lys-349 and Lys-369, in the in-between-ring domain of parkin. ISGylation of these sites promotes parkin's ubiquitin E3 ligase activity by suppressing the intramolecular interaction that maintains its autoinhibited conformation and increases its cytoprotective effect. In conclusion, covalent ISG15 conjugation is a novel mode of modulating parkin activity, and alteration in this pathway may be associated with PD pathogenesis.

## 1. Introduction

Parkinson's disease (PD) is an age-related neurodegenerative disorder that affects 1–2% of adults greater than 60 years of age [1]. Despite extensive research, the cause of PD remains poorly understood. Mutations in several genes responsible for familial PD have been identified, including *SNCA* (encoding α-synuclein), *LRRK2*, *PARK2* (encoding parkin), *PINK1*, *PARK7* (encoding DJ-1) and *ATP13A2* [2].

Parkin is an ubiquitin E3 ligase, which promotes the degradation of target proteins through polyubiquitination or modulates their biochemical properties via mono-ubiquitination [3,4]. Parkin consists of an N-terminal ubiquitin-like (UBL) domain, three RING domains and an in-between-ring (IBR) domain [5]. Most pathogenic mutations of parkin are observed within these domains and are associated with a loss-of-function phenotype [6]. Several parkin substrates have been reported, including Pael receptor, the p38 subunit of aminoacyl-tRNA synthetase complex, ataxin-3, synaptotagmin XI, cyclin E, β-catenin and programmed cell death protein 2 isoform-1 [7]. These substrates accumulate when parkin activity is diminished, consequently contributing to dopaminergic cell death [7]. Therefore, tight regulation of parkin-mediated protein ubiquitination is important for cellular proteolysis and neuronal cell viability.

Recent evidence indicates that parkin is essential for elimination of damaged mitochondria via autophagy [8–10]. Under conditions of mitochondrial depolarization, PINK1 selectively accumulates on the outer membrane of damaged mitochondria, which results in recruitment and phosphorylation of parkin and ubiquitin. PINK1-activated parkin and phosphorylated ubiquitin then promote ubiquitination and subsequent degradation of many mitochondrial proteins, including mitofusion 1 and 2, TOM20, TOM70 and VDACs [8–10]. Parkin-mediated ubiquitination of mitochondrial proteins enhances recruitment of autophagy receptors, such as p62, optineurin and NBR1, resulting in targeting of the damaged mitochondria to LC3-positive autophagosomes for clearance [8–10].

A number of studies have shown that parkin's ubiquitin E3 ligase activity is regulated by various post-translational modifications (PTMs), including phosphorylation, *S*-nitrosylation, sulfhydration, oxidation and dopamine modification [11]. In addition, we have reported that parkin is the conjugation target of multiple UBL modifiers, such as ubiquitin, SUMO and NEDD8. Conjugation to these modifiers affects parkin's activity and/or neuroprotective effects [11–13].

ISGylation is the covalent conjugation of the protein interferon (IFN)-stimulated gene 15 (ISG15) to protein targets. ISG15 was first identified as being induced by type I IFNs [14]. It is composed of two N-terminal UBL domains and a C-terminal Gly–Gly motif, which is critical for target-protein conjugation [15,16]. Like ubiquitination, ISGylation also requires E1, E2 and E3 enzymes, each of which is induced by type I IFNs. UBE1 L is the first and only ISG15-activating enzyme [17]. UbcH8 acts as an ISG15-conjugating enzyme when cells are stimulated with IFN, lipopolysaccharide (LPS) or viral infection [18]. Three ISG15 E3 ligases have been reported: EFP, HHARI and HERC5 [19]. Covalently conjugated ISG15 can be removed from target proteins by UBL-specific isopeptidases, which function by mechanisms similar to those of deubiquitinating enzymes. UBP43, an ISG15 de-conjugating enzyme, cleaves isopeptide bonds between ISG15 and the substrate [20]. ISGylation is associated with various cellular functions, including regulation of JNK and NF-κB signal transduction, ubiquitination and antiviral responses [21–23]. ISG15 also acts as a cytokine, tumour suppressor and oncogenic factor [24–26]. Although more than 300 nascent proteins have been identified as ISGylation targets based on high-throughput proteomics screening [27,28], only a dozen are known to be functionally regulated.

According to the previous reports, low levels of parkin have been detected in blood and immune tissues, including bone marrow, lymph nodes, tonsils and spleen (http://www.proteinatlas.org/ENSG00000185345-PARK2/tissue). In the central nervous system (CNS), parkin is expressed mainly in neurons, but it has also been detected in a small number of glial cells [29], suggesting that parkin may be a novel target of ISGylation. Here, we demonstrate that parkin is modified by ISG15 conjugation when ISGylation components are overexpressed or when cells are treated with type I IFN, LPS or other selected drugs. In addition, ISGylation enhances parkin's ubiquitin E3 ligase activity, consequently increasing its protective effects against cytotoxicity.

## 2. Material and methods

### 2.1. Materials

Dulbecco's modified Eagle's medium (DMEM), fetal bovine serum (FBS), Lipofectamine and PLUS reagents, and Lipofectamine RNAiMAX were purchased from Invitrogen (Carlsbad, CA, USA). Mouse monoclonal anti-FLAG (F3165), rabbit polyclonal anti-FLAG (F7425) and anti-actin (A2066) antibodies; LPS (L3137); camptothecin (CPT; C9911), etoposide (Eto; E1383) and carbonyl cyanide *m*-chlorophenyl hydrazine (CCCP; C2759) were purchased from Sigma-Aldrich (St Louis, MO, USA). Polyclonal anti-parkin antibody (ab15954) and anti-histone H3 antibody (ab1791) were purchased from Abcam (Cambridge, UK). Normal mouse IgG (12–371), normal rabbit IgG (12–370), peroxidase-conjugated mouse IgG (AP124P) and peroxidase-conjugated rabbit IgG (AP132P) were purchased from Millipore (Billerica, MA, USA). Monoclonal anti-parkin antibody (4211; recognizing the C-terminal region surrounding amino acid 400), and anti-VDAC IgG (4866) were purchased from Cell Signaling Technology (Danvers, MA, USA). Mouse anti-Myc (sc-40), rabbit anti-Myc (sc-789), anti-ubiquitin (sc-8017; without ISG15 cross-reactivity), anti-ISG15 (H150; sc-50366), anti-Hsp90 (sc-7949) and anti-tubulin (sc-8035) antibodies and ExactaCruz B (sc-45039) were purchased from Santa Cruz Biotechnology (Santa Cruz, CA, USA). Polyclonal anti-HA (PAB0861) and anti-HERC5 (H00051191-A01) antibodies were obtained from Abnova (Tebu, France), and monoclonal anti-HA antibody (MMS-101P) was purchased from Covance (Dedham, MA, USA). Rabbit anti-ISG15 antibody was kindly provided by C.H. Chung (Seoul National University, Seoul, Korea), and rabbit polyclonal anti-ISG15 antibody (A-600) was purchased from Boston Biochem (Cambridge, MA, USA). MG132 was purchased from A. G. Scientific (San Diego, CA, USA). Protein A-sepharose was purchased from GE Healthcare Life Science (Marlborough, MA, USA). Protein G-agarose 4B resin was purchased from Lugen Sci (Puchon, Gyeonggi-do, Korea). Enhanced chemiluminescence reagent was purchased from PerkinElmer Life and Analytical Sciences (Waltham, MA, USA). IFN-α and -β were purchased from PBL Assay Science (Piscataway, NJ, USA) and Sino Biological (Daxing, China), respectively.

### 2.2. DNA constructs

The mammalian construct encoding Myc-tagged human parkin (pcDNA3.1-Myc-Parkin) was a kind gift from K. Tanaka (Tokyo Metropolitan Institute of Medical Science, Tokyo, Japan). In order to make constructs encoding N-terminal Myc-tagged, HA-tagged or C-terminal V5-tagged parkin, *PARK2* was amplified by PCR and subcloned into vector pRK5-Myc, pRK5-HA or pcDNA3.1-V5-His. To generate truncated parkin mutant constructs, plasmids encoding Myc- or HA-tagged parkin^81–465^, parkin^226–465^, parkin^291–465^, parkin^381–465^ and HA-tagged parkin^1–80^ were generated by PCR amplification of *PARK2*. Each PCR product was subcloned in the pRK5-Myc or pRK5-HA vector. All primers are described in the electronic supplementary material, table S1. Plasmids encoding Myc-tagged human wild-type PINK1 (pBOS-3X-Myc-hPINK1-WT) and its kinase-deficient mutant, with amino acid substitutions K219A, D362A and D384A (pBOS-3X-Myc-hPINK1-KD), were generated as described previously [30]. Plasmids encoding FLAG-tagged wild-type ISG15 and its conjugation-defective AA mutant, and Myc-tagged UbcH8, UBE1 L and wild-type EFP and its catalytically inactive mutant (C13/16S) were kindly provided by C.H. Chung (Seoul National University, Seoul, Korea). The construct encoding HA-tagged UbcH8 (pRK5-HA-UbcH8) was a kind gift from T.M. Dawson (Johns Hopkins University School of Medicine, Baltimore, MD, USA). The plasmid encoding FLAG-tagged HERC5 was kindly provided by K. Hochrainer (Weill Cornell Medical College, New York, NY, USA).

Site-directed mutagenesis was carried out using the QuikChange^®^ XL Site-directed Mutagenesis kit (Stratagene, La Jolla, CA, USA), according to the manufacturer's protocol. The pRK5-HA-parkin and FLAG-tagged HERC5 constructs were amplified by PCR. The plasmids encoding HA-, Myc- or V5-tagged parkin-2KR or Myc-tagged parkin^81–465^-2KR were generated by two rounds of mutagenesis and PCR amplification with combinations of the K349R and K369R primers. Six HA-tagged parkin mutants each having a single PD-associated amino acid substitution (R33Q, R42P, G328E, R334C, T415N or C418R) were produced by mutagenesis of HA-parkin-WT followed by PCR amplification. Primers used in mutagenesis are listed in the electronic supplementary material table S2.

### 2.3. Cell culture and DNA transfection

Human embryonic kidney 293 (HEK293) cells, mouse embryo fibroblast NIH3T3 cells, HeLa cells, mouse microglial BV2 cells and COS-7 cells were maintained in DMEM containing 10% FBS and 100 units ml^−1^ penicillin–streptomycin. Cells were grown at 37°C in a humidified atmosphere of 5% CO_2_. All DNA transfections were performed using Lipofectamine and PLUS reagents according to the manufacturer's protocol.

### 2.4. RNA interference

Small interfering RNA (siRNA) targeting *HERC5* and scrambled control siRNA were designed and synthesized by Bioneer (Seoul, Korea). The sequences of siRNA duplexes are shown in the electronic supplementary material, table S3. HEK293 cells were seeded into six-well plates and transfected with siRNA using Lipofectamine RNAiMAX.

### 2.5. Immunoprecipitation and western blot analysis

To detect protein ISGylation or ubiquitination, cells were rinsed with ice-cold phosphate-buffered saline (PBS) and lysed in RIPA buffer (50 mM Tris (pH 7.4), 150 mM NaCl, 1% Triton X-100, 0.5% sodium deoxycholate, 0.1% SDS and protease inhibitors 0.2 mM phenylmethylsulfonyl fluoride (PMSF), 1 µg ml^−1^ aprotinin, 1 µg ml^−1^ leupeptin, 1 mM Na_3_VO_4_ and 10 mM NaF). For binding assays, cells were lysed in 1% Nonidet P-40 lysis buffer (50 mM Tris (pH 7.4), 150 mM NaCl, 1% Nonidet P-40, 10% glycerol and protease inhibitors 0.2 mM PMSF, 1 µg ml^−1^ aprotinin, 1 µg ml^−1^ leupeptin, 1 mM Na_3_VO_4_ and 10 mM NaF). Cell lysates were clarified by centrifugation at 15 700*g* for 15 min at 4°C. For immunoprecipitation, 1 µg of the appropriate antibody was incubated with cell lysates (0.5–2 mg) overnight at 4°C with gentle rotation. The mixture was incubated for 2 h at 4°C with 30 µl of 1 : 1 protein A-sepharose or protein G-agarose beads, or 40 µl of ExactaCruz B immunoprecipitation mix with gentle rotation. Samples were centrifuged at 9300*g* for 30 s and pellets were washed five times with 1% Nonidet P-40 lysis buffer. Immunocomplexes were dissociated by boiling in SDS-PAGE sample buffer, separated on SDS-PAGE gels and transferred to nitrocellulose membranes. Membranes were blocked for 1 h at room temperature in 25 mM Tris (pH 7.5), 150 mM NaCl and 0.1% Tween® 20 (TBST) containing 5% non-fat dry milk.

### 2.6. Preparation of cytosolic and nuclear fractions

HEK293 cells were scraped from plates in ice-cold PBS and resuspended in hypotonic buffer (10 mM HEPES (pH 7.9), 1.5 mM MgCl_2_, 10 mM KCl, 0.5 mM DTT and protease inhibitor cocktail). Cells were incubated for 10 min on ice and lysed in 1% NP-40, followed by vortexing for 5 s. Cell lysates were centrifuged at 15 700*g* for 5 min at 4°C. The supernatants were considered the cytosolic fractions. Nuclear pellets were washed in hypotonic buffer and resuspended in hypertonic buffer (27 mM HEPES (pH 7.9), 2 mM MgCl_2_, 560 mM NaCl, 270 mM EDTA, 33% glycerol, 0.5 M DTT and protease inhibitor cocktail). Samples were then lysed in 1% Nonidet P-40, followed by vortex-mixing four times for 10 s each. Samples were incubated for 20 min on ice, followed by centrifugation at 15 700*g* for 20 min at 4°C. The supernatants were considered the nuclear fraction.

### 2.7. Preparation of cytosolic and mitochondrial fractions

HEK293 cells were scraped from plates in ice-cold PBS and resuspended in ice-cold digitonin buffer (10 mM PIPES (pH 6.8), 0.015% digitonin, 300 mM sucrose, 100 mM NaCl, 3 mM MgCl_2_, 5 mM EDTA and 1 mM PMSF). Samples were mixed vigorously on a rotating shaker for 10 min at 4°C and centrifuged at 480*g* for 10 min at 4°C. The supernatants were considered the cytosolic fraction. Pellets were washed twice with digitonin buffer and resuspended in ice-cold Triton X-100 buffer (10 mM PIPES (pH 7.4), 0.5% Triton X-100, 300 mM sucrose, 100 mM NaCl, 3 mM MgCl_2_, 5 mM EDTA and 1 mM PMSF). Samples were mixed vigorously on a rotating shaker for 30 min at 4°C and centrifuged at 5000*g* for 10 min at 4°C. The supernatants were considered the mitochondrial fraction.

### 2.8. Phos-tag immunoblotting

After DNA transfection, cells were rinsed in ice-cold PBS and lysed on ice with lysis buffer containing 0.2% Nonidet P-40, 50 mM Tris (pH 7.4), 150 mM NaCl, 10% glycerol, 0.2 mM PMSF, 1 µg ml^−1^ aprotinin, 1 µg ml^−1^ leupeptin, 1 mM Na_3_VO_4_ and 10 mM NaF. Proteins were separated on an 8% gel containing 50 µM Phos-tag (Wako Pure Chemical Industries, Ltd., Japan) and Phos-tag immunoblotting was performed according to the manufacturer's protocol.

### 2.9. Cell viability assays

After transfection with DNA for 24 h, HeLa cells were left untreated or stimulated with IFN-α for an additional 48 h. Medium was removed and Cell Counting Kit-8 (CCK-8; Dojindo Laboratories, Kumamoto, Japan) solution diluted 1 : 20 in media was added to each well. Plates were incubated for 1 h at 37°C, and absorbance at 450 nm was measured using a microplate reader.

### 2.10. Statistical analyses

The statistical significance of cell viability data was determined using unpaired Student's *t*-tests and Sigma Plot v. 9.0. Values are expressed as the mean ± standard error of the mean (s.e.m.).

## 3. Results

### 3.1. HERC5 mediates covalent ISG15 conjugation to parkin in mammalian cells

**Figure 1.**
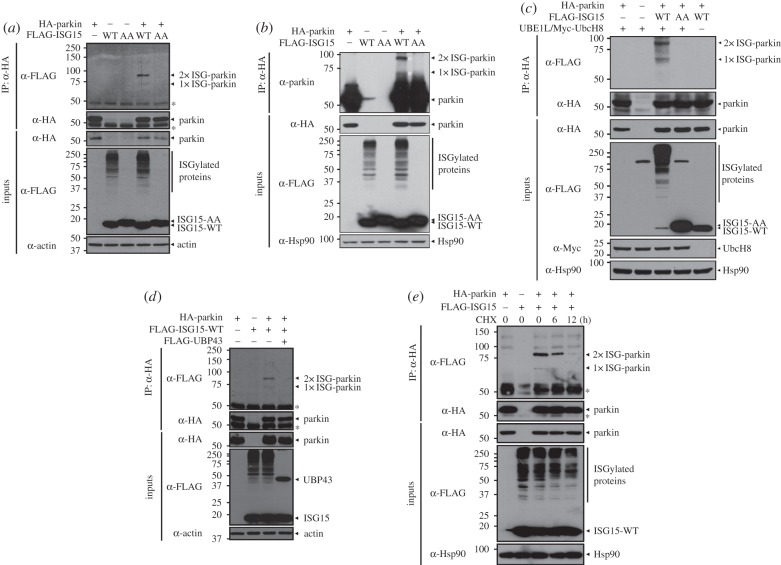
Parkin is a target of ISGylation. (*a,b*) HEK293 cells were transfected with expression vectors encoding HA-tagged parkin, FLAG-tagged wild-type ISG15 (FLAG-ISG15-WT) or its conjugation-defective mutant (FLAG-ISG15-AA) alone or in combination. All samples were also transfected with vectors encoding UBE1 L and Myc-tagged UbcH8 (*a,b,d,e*). Cell lysates were immunoprecipitated with anti-HA antibody, followed by immunoblotting with anti-FLAG (*a*), anti-parkin (*b*) or anti-HA antibody. (*c*) HEK293 cells were transfected with vector encoding HA-parkin, FLAG-ISG15-WT, FLAG-ISG15-AA, pcDNA-UBE1 L or Myc-tagged UbcH8 alone or in combination. Cell lysates were boiled at 100°C for 20 min, immunoprecipitated with anti-HA antibody and immunoblotted with anti-FLAG or anti-HA antibody. (*d*) HEK293 cells were transfected with vectors encoding HA-parkin, FLAG-ISG15-WT or FLAG-USP43 alone or in combination. Cell lysates were immunoprecipitated with anti-HA antibody, followed by immunoblotting with anti-FLAG or anti-HA antibody. (*e*) HEK293 cells were transfected with vector encoding HA-parkin or/and FLAG-ISG15-WT, and treated with 25 µg ml^−1^ cycloheximide (CHX) for the indicated times. Cell lysates were immunoprecipitated with anti-HA antibody and immunoblotted with anti-FLAG or anti-HA antibody. Expression of transiently transfected proteins in cell lysates was monitored by immunoblotting with anti-HA, anti-Myc or anti-FLAG antibody. Actin and Hsp90 served as loading controls. Asterisks indicate IgG heavy chains (*a,d,e*) and white asterisks indicate double (2×)-ISG15-conjugated parkin or single (1×)-ISG15-conjugated parkin (*b*).

First, we examined whether parkin is modified by ISG15 in mammalian cells. As shown in figure 1*a*, two slowly migrating forms of parkin were observed in HEK293 cells co-transfected with expression vectors encoding parkin and wild-type ISG15 (ISG15-WT). These two bands suggest that the upper band is due to conjugation of two ISG15 chains, whereas the lower band corresponds to attachment of a single ISG15 chain. However, these bands were not observed in cells co-expressing parkin and ISG15-AA, the conjugation-defective ISG15 mutant that has the C-terminal Gly–Gly residues substituted with Ala–Ala residues (figure 1*a*). To confirm whether these two bands represent ISG15–parkin conjugates, we performed co-immunoprecipitation assays of cell lysate with anti-HA IgG, followed by immunoblotting with anti-parkin antibody. As shown in figure 1*b*, two identically shifted bands were also observed in lysates of cells overexpressing parkin and ISG15-WT by anti-parkin antibody. To rule out the possibility that parkin is not covalently conjugated to ISG15 but instead binds to intracellular ISGylated proteins, we performed co-immunoprecipitation after cell lysates were boiled at 100°C. Under these conditions, the shifted bands were still observed in the cell lysates of cells expressing HA-parkin and FLAG-ISG15-WT (figure 1*c*). In addition, these two shifted bands selectively disappeared in the presence of co-expressed UBP43 (figure 1*d*).

Based on the previous report that the principal targets of ISG15 conjugation are nascent polypeptide chains [31], we next analysed parkin ISGylation after cycloheximide treatment of cells. As shown in figure 1*e*, ISGylated parkin levels decreased after cycloheximide treatment in a time-dependent manner. These results suggest that newly synthesized parkin is a target of covalent ISG15 conjugation.

**Figure 2.**
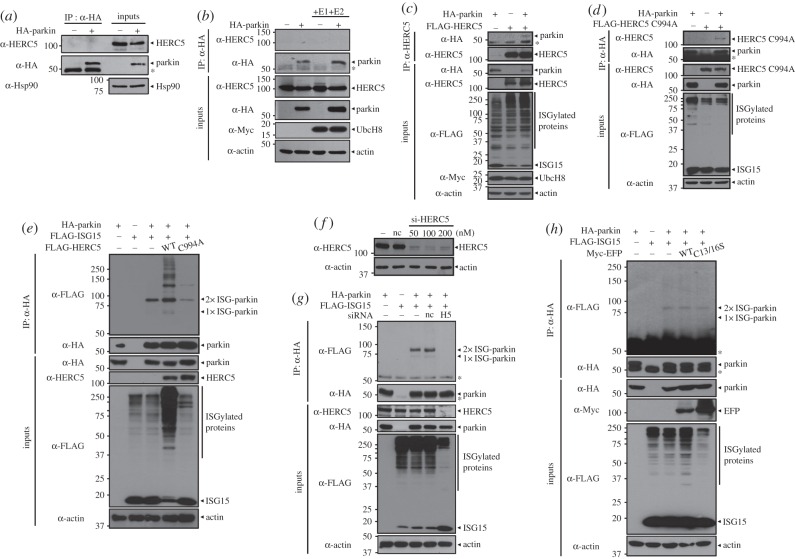
HERC5 is an essential E3 ISG15 protein ligase of parkin. (*a*) HEK293 cells were mock transfected or transfected with expression vector encoding HA-tagged parkin. Cell lysates were immunoprecipitated with anti-HA antibody, followed by immunoblotting with anti-HERC5 or anti-HA antibody. (*b*) HEK293 cells were transfected with expression vector encoding HA-parkin, pcDNA-UBE1 L or Myc-tagged UbcH8 alone or in combination. Cell lysates were immunoprecipitated with anti-HA antibody, followed by immunoblotting with anti-HERC5 or anti-HA antibody. (*c,d*) Where specified, HEK293 cells were transfected with expression vector encoding FLAG-tagged wild-type HERC5 (FLAG-HERC5-WT) (*c*) or its catalytically inactive form (FLAG-HERC5-C994A) (*d*). Cell lysates were immunoprecipitated with anti-HA antibody, followed by immunoblotting with anti-HERC5, anti-HA or anti-FLAG antibody. (*e*) HEK293 cells were transfected with expression vector encoding HA-parkin, FLAG-ISG15-WT, FLAG-HERC5-WT or FLAG-HERC5-C994A alone or in combination. Cell lysates were immunoprecipitated with anti-HA antibody, followed by immunoblotting with anti-FLAG or anti-HA antibody. (*f*) HEK293 cells were mock transfected (−) or transfected for 48 h with non-specific control siRNA (nc; 200 nM) or the indicated concentration of *HERC5*-specific siRNA (si-*HERC5*). Cell lysates were immunoblotted with anti-HERC5 antibody. (*g*) HEK293 cells were transfected for 48 h with nc siRNA (50 nM) or si-*HERC5* (H5; 50 nM). Where indicated, cells were additionally transfected for 24 h with vector encoding HA-parkin or FLAG-ISG15-WT alone or in combination. Cell lysates were immunoprecipitated with anti-HA antibody, followed by immunoblotting with anti-FLAG or anti-HA antibody. (*h*) HEK293 cells were transfected with vector encoding HA-parkin, FLAG-ISG15-WT, Myc-EFP-WT or Myc-EFP-C13/16S alone or in combination. Cell lysates were immunoprecipitated with anti-HA antibody, followed by immunoblotting with anti-FLAG or anti-HA antibody. All samples were also transfected with vector encoding FLAG-ISG15-WT, UBE1 L, Myc-UbcH8 and HA-parkin (*c*,*d*) or UBE1 L and Myc-tagged UbcH8 (*e*,*g*,*h*). Expression of transiently transfected proteins was monitored by immunoblotting of cell lysates with anti-HERC5, anti-HA, anti-Myc or anti-FLAG antibody. Actin and Hsp90 served as loading controls. Asterisks indicate IgG heavy chains.

To further examine whether the action of a specific ISG15-E3 ligase is required for parkin ISGylation, we tested the interaction between parkin and HERC5. As shown in figure 2*a–d*, parkin specifically binds to wild-type HERC5 (HERC5-WT) and its catalytically inactive mutant (HERC5-C994A) when the proteins were co-expressed with the three components of the ISG15 conjugation system (i.e. ISG15, UBE1 L and UbcH8). These results indicate that the interaction between HERC5 and parkin might be mediated by another, yet unknown, component. Next, we tested whether HERC5 acts as an ISG15 E3 ligase to promote parkin ISGylation. As shown in figure 2*e*, wild-type HERC5 markedly enhanced the ISGylation of parkin, whereas HERC5-C994A had no effect. To confirm the specific effect of HERC5 on parkin ISGylation, we evaluated the effect of siRNA-mediated *HERC5* knockdown on parkin ISGylation (figure 2*f*). When cells were treated with siRNA targeting *HERC5*, the two parkin–ISG15 conjugate bands were abolished (figure 2*g*). However, parkin ISGylation was unaffected by treatment with control siRNA (figure 2*g*). Lastly, we tested whether another ISG15 E3 ligase, EFP [19], promotes parkin ISGylation. Unlike HERC5, EFP has no effect on parkin ISGylation (figure 2*h*). These results demonstrate that HERC5 is a specific ISG15 E3 ligase of parkin.

### 3.2. ISG15 is conjugated to the Lys349 and Lys369 residues of parkin

**Figure 3.**
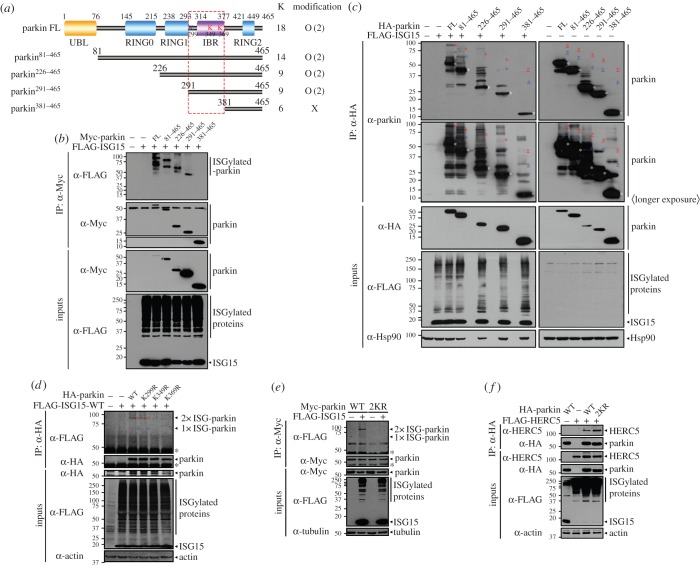
Identification of parkin ISGylation sites. (*a*) Schematic diagram of full-length (FL) parkin and its truncated mutants. The parkin ISGylation regions are outlined by the red box. The blue and red ‘K's represent the three lysine residues located in the 291–380 amino acid region of parkin. The two red ‘K's indicate the exact ISGylation sites. (*b*,*c*) HEK293 cells were transfected with constructs encoding Myc- (*b*) or HA-tagged (*c*) parkin-FL or its truncated mutants (i.e. parkin^81–465^, parkin^226–465^, parkin^291–465^ or parkin^381–465^), or FLAG-ISG15-WT alone or in combination. Cell lysates were immunoprecipitated with anti-Myc (*b*) or anti-HA (*c*) antibodies, followed by immunoblotting with anti-FLAG (*b*), anti-parkin (*c*) or anti-Myc antibody. Red, blue (*c,d*) and white (*c*) asterisks indicate double (2×)-, single (1×)-ISG15-conjugated and unmodified parkin, respectively. Red underlined asterisks indicate predicted double-ISG15-conjugated parkin, and blue underlined asterisks indicate predicted single-ISG15-conjugated parkin (*c*). (*d*) HEK293 cells were transfected with constructs encoding HA-parkin-WT or its point mutants (HA-parkin-K299R, HA-parkin-K349R and HA-parkin-K369R) or FLAG-ISG15-WT alone or in combination. Cell lysates were immunoprecipitated with anti-HA antibody, followed by immunoblotting with anti-FLAG or anti-HA antibody. (*e*) HEK293 cells were transfected with vector encoding Myc-parkin-WT or Myc-parkin-K349R/K369R mutant (2KR) and FLAG-ISG15-WT alone or in combination. Cell lysates were immunoprecipitated with anti-Myc antibody, followed by immunoblotting with anti-FLAG or anti-Myc antibody. (*f*) HEK293 cells were transfected with vector encoding HA-parkin-WT, HA-parkin-2KR or FLAG-HERC5 alone or in combination. Cell lysates were immunoprecipitated with anti-HA antibody, followed by immunoblotting with anti-HERC5 or anti-HA antibody. Expression of transiently transfected proteins was monitored by immunoblotting of cell lysates with anti-Myc, anti-HA or anti-FLAG antibody. All samples were also transfected with UBE1 L and HA-tagged (*b*,*e*) or Myc-tagged UbcH8 (*c*,*d*,*f*). Actin, Hsp90 and tubulin served as loading controls. Black asterisks indicate IgG heavy chains (*b*,*d*,*e*).

To determine the exact ISGylation site(s) within parkin, several truncated parkin mutants (parkin^81–465^, parkin^226–465^, parkin^291–465^ and parkin^381–465^) were examined to determine whether they were targets of ISGylation (figure 3*a*). Co-immunoprecipitation assays revealed that parkin^81–465^ and parkin^226–465^ fragments were ISGylated to the same degree as wild-type parkin (figure 3*b,c*). In addition, parkin^291–465^ was the target of greatly reduced double-ISG15 conjugation, but of a still significant level of mono-ISG15 conjugation. However, substantial ISGylation was not observed with the parkin^381–465^ mutant (figure 3*b,c*). These results indicate that the region of amino acids 291–380 of parkin is critical for ISGylation.

Similar to ubiquitin, the C-terminal Gly–Gly residue of ISG15 is conjugated to the ε-amine group of lysine (K) residues on the substrate. Because three lysine residues (K299, K349 and K369) are present in the region of amino acid residues 291–380 of parkin, we next determined which lysine residue is the targeting site of ISGylation. To map the exact ISGylation site(s), we generated three parkin mutants, each with substitution of arginine for one of the lysines (K299R, K349R and K369R) and then tested the effect of the substitutions on parkin ISGylation. The K299R mutant was ISGylated to an extent similar to that of wild-type parkin (figure 3*d*). By contrast, a significant level of ISGylation of the parkin-K349R and -K369R mutants was not observed. Based on our previous finding that two ISG15 moieties can attach to parkin, these data suggest that K349 and K369 are the specific parkin ISGylation sites. To confirm this hypothesis, we created parkin mutant 2KR in which both K349 and K369 were substituted with arginine. Co-immunoprecipitation assays revealed that ISGylation bands were completely abolished in the 2KR mutant compared with wild-type parkin (figure 3*e*). To assess the specificity of these lysine residues for HERC5 binding, we performed co-immunoprecipitation assays of lysates of cells co-expressing wild-type parkin or the 2KR mutant and HERC5. As shown in figure 3*f*, the parkin-2KR mutant interacts with HERC5, indicating that residues K349 and K369 are important for ISG15 conjugation, but do not affect the binding of HERC5.

Taken together, our data suggest that the residues K349 and K369 within the parkin IBR domain are the sites for covalent ISG15 conjugation.

### 3.3. ISGylation does not affect the localization of parkin

**Figure 4.**
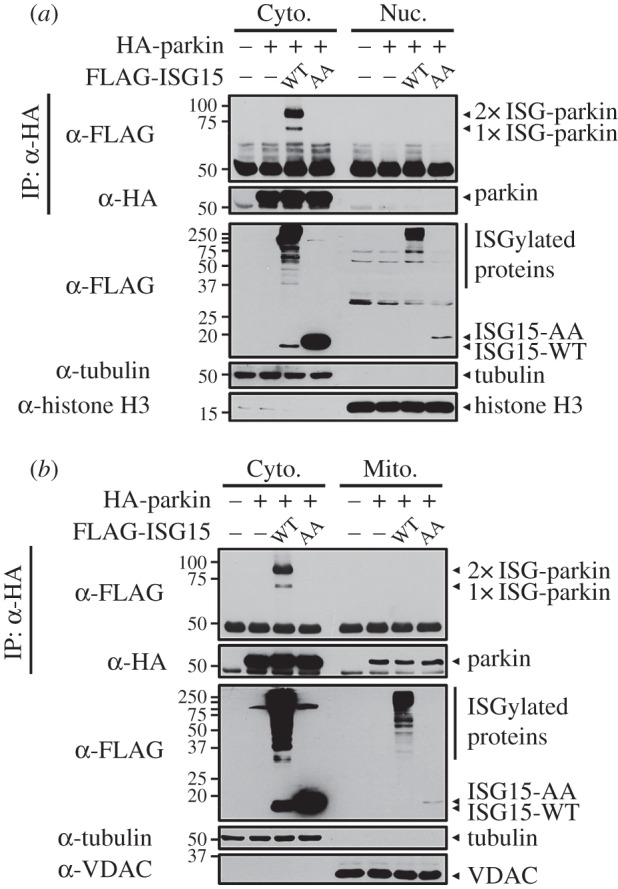
ISGylation does not change the intracellular localization of parkin. (*a*,*b*) Where specified, HEK293 cells were co-transfected for 24 h with vector encoding UBE1 L plus Myc-UbcH8 together with HA-parkin-WT, FLAG-ISG15-WT or FLAG-ISG15-AA alone or in combination. Cell lysates were separated into cytosolic and nuclear fractions (*a*) or cytosolic and mitochondrial fractions (*b*). Samples were then immunoprecipitated with anti-HA antibody, followed by immunoblotting with anti-FLAG or anti-HA antibody. Tubulin served as a marker for the cytosolic fraction, histone H3 as a marker for the nuclear fraction and VDAC as a marker for the mitochondrial fraction.

We next investigated whether ISGylation affects the biochemical and functional properties of parkin. Although parkin is primarily localized in the cytoplasm in the resting state [32], it moves into the nucleus or mitochondria upon non-covalent protein interactions or PTMs [12,33]. Based on these findings, we first evaluated whether ISGylation changes the subcellular localization of parkin. Consistent with previous findings, overexpression of wild-type parkin resulted in predominantly cytoplasmic localization (figure 4*a,b*). Moreover, co-expression of wild-type parkin and ISG15 does not trigger translocation of cytosolic parkin into the nuclear or mitochondrial fraction (figure 4*a,b*), suggesting that parkin localization is unaffected by ISG15 conjugation. Overall, although we could not clarify the intracellular location of ISG15-conjugated parkin directly, these data suggest that ISGylation does not affect parkin subcellular localization.

### 3.4. ISGylation increases the ubiquitin E3 ligase activity of parkin

**Figure 5.**
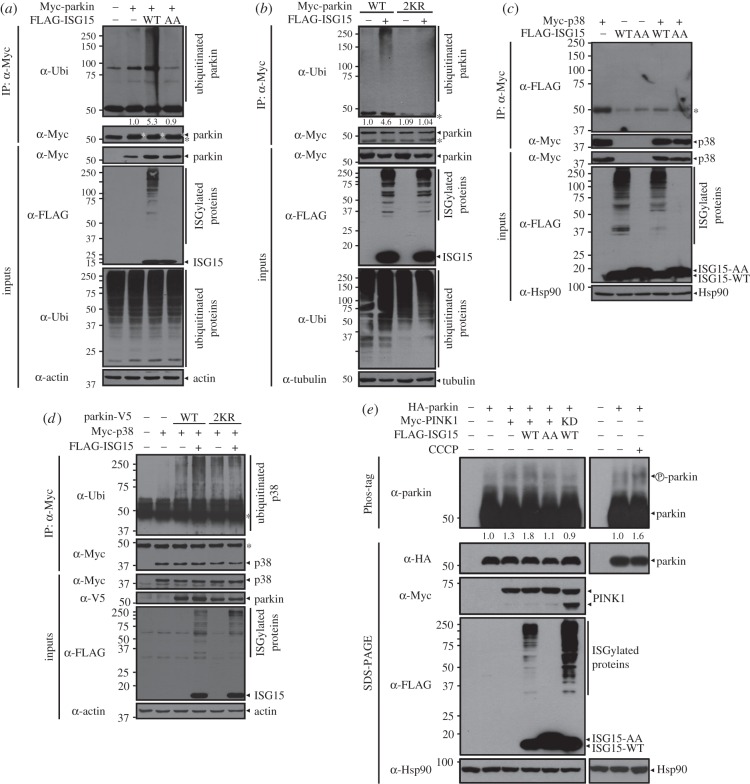
ISGylation promotes parkin E3 ubiquitin ligase activity. (*a*) HEK293 cells were transfected for 24 h with vector encoding Myc-parkin, FLAG-ISG15-WT or FLAG-ISG15-AA alone or in combination. Cells were treated for 6 h with 10 µM MG132. Cell lysates were immunoprecipitated with anti-Myc antibody, followed by immunoblotting with anti-ubiquitin (Ubi) or anti-Myc antibody. White asterisks indicate immunoprecipitated parkin. (*b*) HEK293 cells were transfected for 24 h with vectors encoding Myc-parkin-WT, Myc-parkin-2KR or FLAG-ISG15-WT alone or in combination. Cells were treated for 6 h with 10 µM MG132. Cell lysates were immunoprecipitated with anti-Myc antibody, followed by immunoblotting with anti-ubiquitin (Ubi) or anti-Myc antibody. The values at the bottom of the top panel indicate the relative intensities of parkin autoubiquitination bands measured using MultiGauge v. 3.1 (*a*,*b*). (*c*) Where indicated, HEK293 cells were co-transfected with vectors encoding UBE1 L plus HA-UbcH8 together with Myc-p38, FLAG-ISG15-WT or FLAG-ISG15-AA alone or in combination. Cell lysates were immunoprecipitated with anti-Myc antibody, followed by immunoblotting with anti-FLAG or anti-Myc antibody. Expression of transiently transfected proteins was monitored by immunoblotting of cell lysates with anti-Myc or anti-FLAG antibody. (*d*) HEK293 cells were transfected for 24 h with vectors encoding Myc-p38, parkin-WT-V5, parkin-2KR-V5 or FLAG-ISG15-WT alone or in combination. Cells were treated for 6 h with 10 µM MG132. Cell lysates were immunoprecipitated with anti-Myc antibody, followed by immunoblotting with anti-ubiquitin (Ubi) or anti-Myc antibody. Expression of transiently transfected proteins was monitored by immunoblotting of cell lysates with anti-Myc, anti-V5 or anti-FLAG antibody. (*e*) HEK293 cells were transfected for 24 h with vectors encoding HA-parkin, Myc-PINK1-WT or its kinase-deficient mutant (KD), FLAG-ISG15-WT, or FLAG-ISG15-AA alone or in combination. Cells were left untreated or treated for an additional 6 h with 10 µM CCCP. Cell lysates were resolved by electrophoresis through a Phos-tag gel, followed by immunoblotting with anti-parkin antibody (Phos-tag). Expression of transfected proteins was identified in cell lysates with anti-HA, anti-Myc or anti-FLAG antibody (SDS-PAGE). All samples were also transfected with vectors encoding UBE1 L and HA-tagged UbcH8. Actin, Hsp90 and tubulin served as loading controls. Asterisks indicate IgG heavy chains.

We next examined whether ISGylation affects the ubiquitin E3 ligase activity of parkin. Parkin activity was measured using autoubiquitination assays. As shown in figure 5*a*, parkin autoubiquitination was increased in the presence of wild-type ISG15. This effect was not observed in the presence of the conjugation-defective ISG15-AA mutant. Next, we determined whether parkin ISGylation on the K349 and K369 residues enhanced its E3 ubiquitin ligase activity. Compared with wild-type parkin, autoubiquitination of the parkin-2KR mutant was significantly reduced in the ISG15-overexpressing cells (figure 5*b*). Although parkin is self-modified by multiple mono-ubiquitination events, the specific ubiquitination sites have not been identified [4,34]. Therefore, the K349 and K369 residues are likely to be dual targets of ubiquitination and ISGylation; thus, mutation of these sites might affect autoubiquitination of parkin. However, because the pattern of parkin mutant 2KR autoubiquitination was identical to that of wild-type parkin (figures 5*b* and 8*b*), these two sites are likely to be specifically modified by ISGylation, not ubiquitination.

Previous reports revealed that several PTMs regulate the ubiquitin E3 ligase activity of parkin, thus modulating steady state levels of target proteins, such as p38, TRAF2 and Mfn1/2, through ubiquitination [7]. To verify the positive regulatory effect of ISGylation on parkin activity, we investigated whether ISGylation affects ubiquitination of the parkin target-protein p38. As ISGylation can also alter ubiquitination [26], we tested whether p38 itself could be modified by ISG15 conjugation, as a control. Co-immunoprecipitation assays showed no apparent ISGylated p38 bands in the presence of ISG15 (figure 5*c*). In addition, when we tested the effect of parkin on p38 ubiquitination, it was greatly increased by wild-type parkin but not by its conjugation-resistant mutant. We then examined the effect of parkin on changes in p38 ubiquitination. Ubiquitination of p38 was enhanced in cells co-expressing wild-type parkin and ISG15 compared with cells expressing wild-type parkin alone (figure 5*d*). However, p38 ubiquitination was similar in cells co-expressing parkin mutant 2KR and ISG15 and those expressing the 2KR mutant alone (figure 5*d*).

Recently, several studies demonstrated that activation and recruitment of parkin to damaged mitochondria involves PINK1-mediated phosphorylation of both parkin and ubiquitin during mitophagy [35–37]. Based on these findings, we next investigated whether ISGylation affects PINK1-mediated parkin phosphorylation. Analyses of cell lysates by electrophoresis through Phos-tag gels revealed that PINK1 directly phosphorylates parkin as in cells treated with carbonyl cyanide *m*-chlorophenyl hydrazone (CCCP), a potent mitochondrial oxidative phosphorylation uncoupler. In addition, parkin phosphorylation by wild-type PINK1 was greatly increased in the presence of wild-type ISG15, but not with the conjugation-defective ISG15-AA mutant (figure 5*e*)*.* Moreover, this effect was not seen with the PINK1 kinase-deficient mutant, verifying that phosphorylation of parkin is specifically mediated by the action of PINK1 (figure 5*e*).

Taken together, our data suggest that ISGylation positively regulates the ubiquitin E3 ligase activity of parkin.

### 3.5. ISGylation inhibits parkin's intramolecular interaction

**Figure 6.**
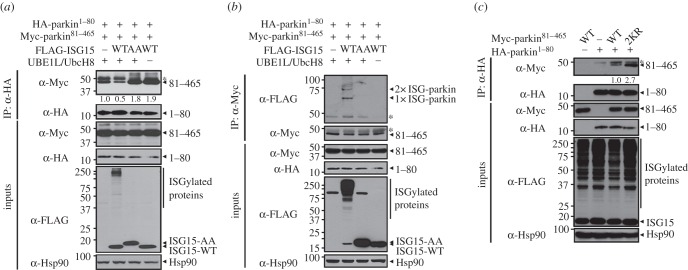
ISGylation inhibits intramolecular autoinhibitory interaction of parkin. (*a*,*b*) HEK293 cells were transfected for 24 h with vectors encoding HA-parkin^1–80^ and Myc-parkin^81–465^ alone or together with FLAG-ISG15-WT, FLAG-ISG15-AA, pcDNA-UBE1 L or Myc- (*a*) or HA-tagged (*b*) UbcH8, as indicated. Cell lysates were immunoprecipitated with the anti-HA (*a*) or anti-Myc (*b*) antibodies, followed by immunoblotting with anti-Myc (*a*) or anti-FLAG (*b*) antibodies. Hsp90 served as a loading control and asterisks indicate IgG heavy chains. (*c*) HEK293 cells were transfected for 24 h with vectors encoding HA-parkin^1–80^, Myc-parkin^81–465^-WT or Myc-parkin^81–465^-2KR alone or in combination. Cell lysates were immunoprecipitated with anti-HA antibody, followed by immunoblotting with anti-Myc antibody. All lanes show overexpression of FLAG-tagged ISG15, pcDNA-UBE1 L and Myc-tagged UbcH8. Hsp90 served as a loading control and asterisks indicate IgG heavy chains. The values at the bottom of the top panel (*a*,*c*) indicate the relative intensities of intramolecular interaction bands measured using the MultiGauge software (v. 3.1). The intramolecular interaction bands were normalized to bands immunoprecipitated with anti-HA antibody.

We next investigated how ISGylation enhances the ubiquitin E3 ligase activity of parkin. According to previous report, native parkin exists in a closed conformation via an intramolecular autoinhibitory interaction between the UBL domain and the PUB motif [38]. Therefore, we examined the effect of parkin ISGylation on this intramolecular interaction. Owing to the technical difficulty inherent in efficiently detecting an intramolecular protein interaction, we generated two plasmids encoding N-terminal parkin, parkin^1–80^ including the UBL domain and parkin^81–465^ including the PUB motif, and measured the effect of ISG15 conjugation on their putative binding. Co-immunoprecipitation assays revealed that cells expressing the two fragments alone showed binding between parkin^1–80^ and parkin^81–465^ (figure 6*a*), confirming the intramolecular interaction and the validity of our detection method. Moreover, the intramolecular interaction between the parkin^1–80^ and parkin^81–465^ fragments was significantly inhibited when cells coexpressed ISG15-WT, E1 and E2. This effect was not observed in the ISG15-AA mutant-overexpressing cells and in the ISG15-WT only overexpressing cells (figure 6*a*). To confirm that the intramolecular interaction of parkin is inhibited by ISGylation, we examined whether the parkin^81–465^ fragment was a target of ISG15 conjugation under the same assay conditions as in figure 6*a*. Co-immunoprecipitation analyses revealed that the parkin^81–465^ fragment was highly modified by ISG15 conjugation (figure 6*b*). Next, we determined whether parkin ISGylation on the K349 and K369 residues inhibit its intramolecular interaction. Compared with lysates of cells expressing the wild-type parkin^81–465^ fragment, the intramolecular interaction of parkin^81–465^–2KR was significantly increased (figure 6*c*). These data demonstrate that ISGylation suppresses the intramolecular interaction that maintains parkin's autoinhibited conformation, which may underlie the ability of ISG15 conjugation to stimulate parkin activity.

### 3.6. Type I interferon enhances parkin E3 ubiquitin ligase activity

**Figure 7.**
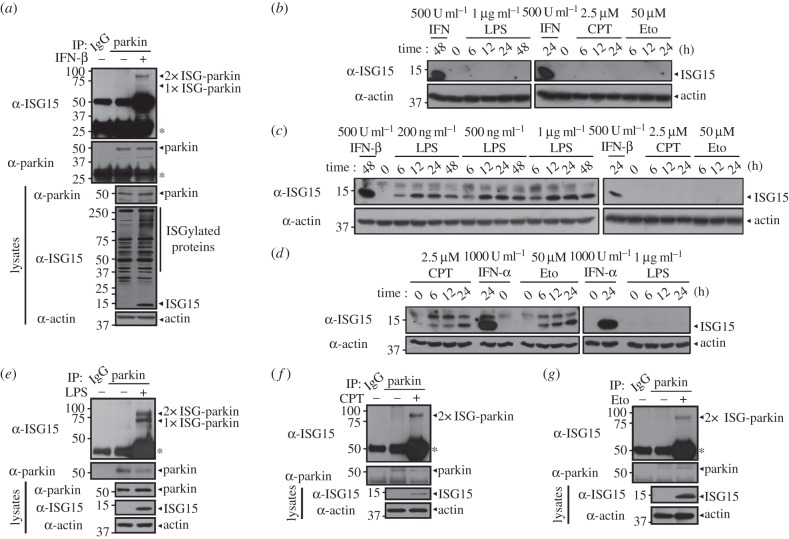
ISGylation of parkin is induced by various signalling pathways. (*a*) NIH3T3 cells were treated for 48 h with vehicle or 500 U ml^−1^ IFN-β. Cell lysates were immunoprecipitated with pre-immune IgG or anti-parkin antibody, followed by immunoblotting with anti-ISG15 or anti-parkin antibody. (*b–d*) Where specified, NIH3T3 (*b*), BV2 (*c*) or COS-7 (*d*) cells were treated with 500 U ml^−1^ IFN-β; 1000 U ml^−1^ IFN-α; 200 ng ml^−1^, 500 ng ml^−1^ or 1 µg ml^−1^ lipopolysaccharide (LPS); 2.5 µM camptothecin (CPT) or 50 µM etoposide (Eto) for the indicated times. Cell lysates were subjected to immunoblotting with anti-ISG15 IgG. (*e*) BV2 cells were treated for 24 h with vehicle or 1 µg ml^−1^ LPS. Cell lysates were immunoprecipitated with pre-immune IgG or anti-parkin antibody, followed by immunoblotting with anti-ISG15 or anti-parkin antibody. (*f*,*g*) COS-7 cells were treated for 24 h with vehicle or 2.5 µM CPT (*f*) or 50 µM Eto (*g*). Cell lysates were immunoprecipitated with pre-immune IgG or anti-parkin antibody, followed by immunoblotting with anti-ISG15 or anti-parkin antibody. Actin served as a loading control. Asterisks indicate IgG heavy or light chains.

Type I IFNs (IFN-α and -β) are essential components of the innate antiviral response [39]. The JAK family of tyrosine kinases is activated when IFNs bind to cell surface receptors, resulting in receptor dimerization [40]. Activated JAKs phosphorylate STAT proteins, and STAT proteins bind IFN response elements at the promoters of IFN-stimulated genes, including *ISG15* [41]. Therefore, treatment with type I IFNs induces the accumulation of ISG15 and its conjugating enzyme system, which subsequently increases ISGylation of target proteins. We next investigated whether parkin is a target of ISGylation in response to type I IFN treatment. Based on a report that treatment with type I IFN induces protein ISGylation in NIH3T3 cells [19], co-immunoprecipitation analyses of NIH3T3 cells showed that endogenous parkin was ISGylated after treatment with IFN-β (figure 7*a*) as well as with IFN-α (data not shown).

As ISG15 is strongly induced by various types of external stimuli, such as virus infection, LPS, genotoxic stresses and retinoic acid [42–45], we then investigated whether ISG15 conjugation to parkin occurs in response to other selected signals, such as LPS, camptothecin (CPT) and etoposide (Eto). First, we examined whether expression of ISG15 is induced by LPS, CPT and Eto in various cell types. As shown in figure 7*b*, these stimuli, but not IFN-β, failed to induce ISG15 in NIH3T3 cells. However, BV2 cells treated with LPS and IFN-β showed specific induction of ISG15, whereas COS-7 cells showed induction of ISG15 in response to CPT, Eto and IFN-β (figure 7*c,d*). We then determined whether parkin is modified by some of these ISG15 inducers. As shown in figure 7*e–g*, endogenous parkin was ISGylated in response to LPS in BV2 cells, and to CPT and Eto in COS-7 cells. These results suggest the parkin ISGylation occurs in response to selected stimuli in specific cell types.

**Figure 8.**
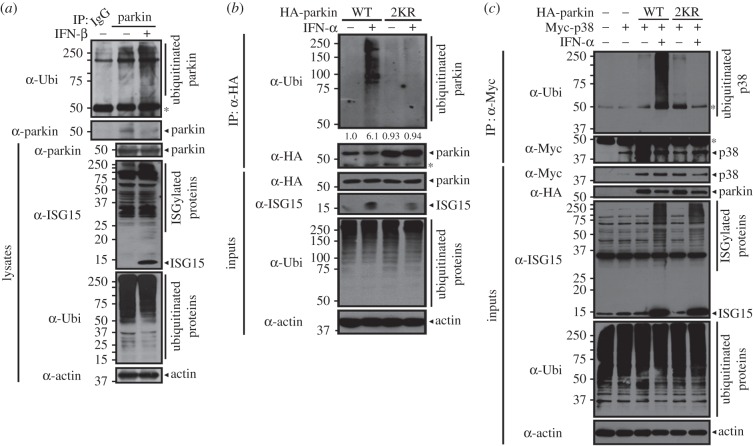
Type I IFN treatment stimulates E3 ubiquitin ligase activity of parkin. (*a*) NIH3T3 cells were treated for 48 h with vehicle or 500 U ml^−1^ IFN-β and cultured for an additional 6 h in the presence of 10 µM MG132. Cell lysates were immunoprecipitated with IgG or anti-parkin antibodies, followed by immunoblotting with anti-ubiquitin (Ubi) or anti-parkin antibody, as indicated. (*b*) HeLa cells were transfected for 24 h with vector encoding HA-parkin-WT or HA-parkin-2KR. Cells were left untreated or treated for 48 h with 1000 U ml^−1^ IFN-α, and cultured for an additional 6 h in the presence of 10 µM MG132. Cell lysates were immunoprecipitated with anti-HA antibody, followed by immunoblotting with anti-ubiquitin (Ubi) or anti-HA antibody. The values at the bottom of the top panel indicate the relative intensities of parkin autoubiquitination bands measured using MultiGauge v. 3.1. (*c*) HeLa cells were transfected for 24 h with vectors encoding Myc-p38, HA-parkin-WT or HA-parkin-2KR alone or in combination. Cells were left untreated or treated for 48 h with 1000 U ml^−1^ IFN-α and cultured for an additional 6 h in the presence of 10 µM MG132, as indicated. Cell lysates were immunoprecipitated with anti-Myc antibody, followed by immunoblotting with anti-ubiquitin (Ubi) or anti-Myc antibody. Actin served as a loading control. Asterisks indicate IgG heavy chains.

We next investigated whether type I IFN treatment regulates parkin ubiquitin E3 ligase activity through ISGylation. As shown in figure 8*a*, parkin autoubiquitination was enhanced in cells treated with IFN-β (figure 8*a*). Next, we evaluated the effect of mutating the parkin–ISG15 conjugation sites K349 and K369 on parkin's ubiquitin E3 ligase activity after type I IFN treatment. For these assays, we utilized HeLa cells, which express no endogenous parkin [46], rather than NIH3T3 cells. As shown in figure 8*b*, parkin autoubiquitination was significantly increased upon IFN-α treatment. However, this increase was not observed in the presence of the parkin-2KR mutant (figure 8*b*). Finally, we examined the effect of the parkin-2KR mutant on p38 ubiquitination after IFN-α stimulation. Assays of p38 ubiquitination demonstrated that IFN-α treatment, and subsequent increase in parkin ISGylation, also promotes p38 ubiquitination (figure 8*c*). Accordingly, this effect was greatly diminished in the presence of the parkin-2KR mutant (figure 8*c*). These results suggest that type I IFN treatment enhances parkin E3 ubiquitin ligase activity through ISGylation.

### 3.7. ISGylation promotes the cytoprotective effect of parkin

**Figure 9.**
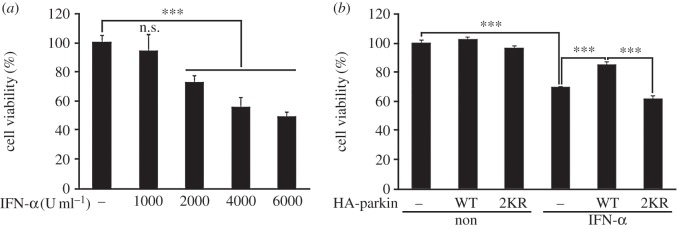
Parkin ISGylation enhances its cytoprotective effect against IFN-α toxicity. (*a*) HeLa cells were treated for 48 h with vehicle (−) or the indicated concentrations of IFN-α, and cell viability was assessed using CCK-8 assay. Data are represented as the mean ± s.e.m. of three-independent experiments (***p < 0.0005). (*b*) Where indicated, HeLa cells were mock transfected (−) or transfected for 24 h with vector encoding HA-parkin-WT or HA-parkin-K349R/K369R (2KR). Cells were left untreated (non) or treated for 48 h with 4000 U ml^−1^ IFN-α. Cell viability was assessed using CCK-8 assay. Data are represented as the mean ± s.e.m. of three independent experiments (***p < 0.0001).

Based on the finding that parkin has cytoprotective activity against cell death induced by various toxic agents, including 6-OHDA, H_2_O_2_ and rotenone [47], we examined whether ISG15 conjugation affects the cell-protective actions of parkin after treatment with IFN-α at concentrations high enough to trigger cytotoxic effects. First, after HeLa cells were treated with various concentrations of IFN-α, we measured cell viability. As shown in figure 9*a*, HeLa cell viability decreased dose-dependently in the presence of IFN-α, reaching its maximum effect at 4000 U ml^−1^. We further examined whether ISGylation affects the cytoprotective activity of parkin. As expected, HeLa cells transfected with wild-type parkin showed much higher viability in the presence of 4000 U ml^−1^ IFN-α (figure 9*b*). In addition, the cytoprotective effect against IFN-α was much lower in cells expressing the parkin-2KR mutant (figure 9*b*). These results demonstrate that ISG15 conjugation enhances the protective function of parkin against IFN-α-induced cell death through K349 and K369 modifications.

### 3.8. Some familial Parkinson's disease-associated missense mutations of parkin display defective ISGylation

**Figure 10.**
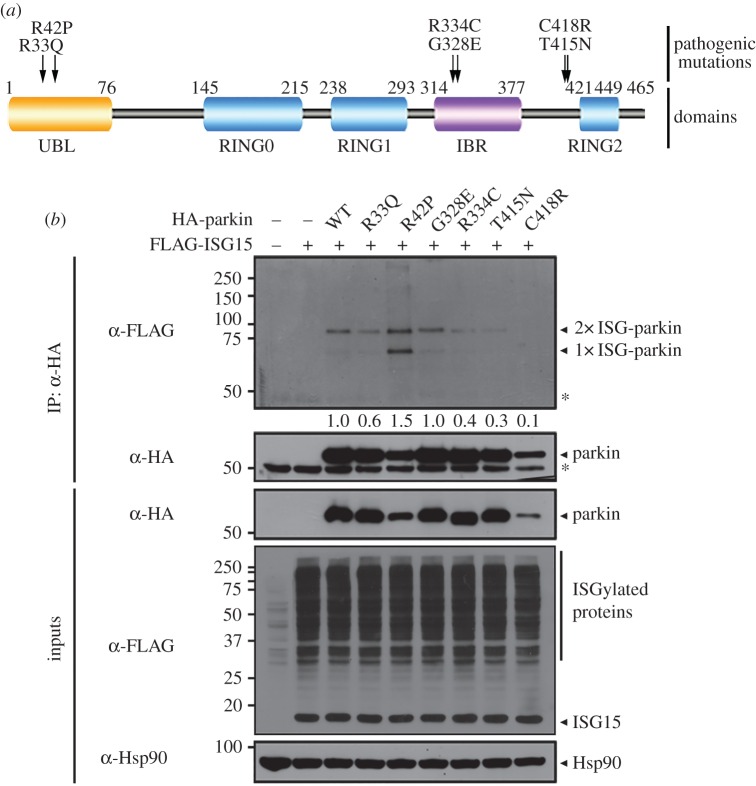
Some PD-associated missense mutations affect parkin ISGylation. (*a*) Schematic diagram of the six parkin mutants each having a single PD-associated missense mutation (R33Q, R42P, G328E, R334C, T415N and C418R), used for ISGylation assay. (*b*) HEK293 cells were transfected with vector encoding HA-tagged wild-type parkin (WT) or its point mutants, or FLAG-ISG15-WT alone or in combination. Cell lysates were immunoprecipitated with anti-HA antibody, followed by immunoblotting with anti-FLAG or anti-HA antibody. Hsp90 served as a loading control. Asterisks indicate IgG heavy chains. The values at the bottom of the top panel (*b*) indicate the relative intensities of 2× ISG-parkin bands, which were measured using MultiGauge software (v. 3.1). The 2× ISG-parkin bands were normalized to bands immunoprecipitated with anti-HA antibody.

Lastly, we investigated whether the effect of six PD-linked missense *parkin* mutations (R33Q, R42P, G328E, R334C, T415N and C148R) located in nearby ISGylation sites (IBR domain) or autoinhibition-related regions (UBL domain and PUB motif) on parkin ISGylation (figure 10*a,b*). Among the mutations, ISGylation was decreased by more than 70% when mutations occurred in the PUB motif (T415N and C418R). In addition, ISGylation was decreased by more than 40% when the mutation occurred in the UBL (R33Q) or IBR domain (R334C) (figure 10*b*). However, the extent of parkin ISGylation was increased by more than 50% with the R42P mutant, whereas the G328E mutant showed an almost identical extent of ISG15 conjugation as wild-type parkin (figure 10*b*). These results suggest that the effect of cytotoxicity by some *parkin* loss-of-function mutations might be triggered through dysregulation of parkin ISGylation, which may also play a role during PD progression.

## 4. Discussion

Although ubiquitin is the best studied post-translational modifier, there is a growing family of UBL proteins that modify cellular targets in pathways that are parallel to, but distinct from, the ubiquitin pathway. These proteins include SUMO, NEDD8, ISG15, FAT10 and Hub1, and have novel functions and influence diverse biological processes. Regarding the link between UBL modification and PD pathogenesis, SUMO-1 expression is increased in the Lewy bodies of PD patient brains as well as in the unilateral rotenone-lesioned mouse model of PD [48,49]. NEDD8 accumulation has also been observed in neuronal and glial inclusions in neurodegenerative disorders [50,51]. Moreover, several familial gene products are modified by UBLs. For example, α-synuclein and DJ-1 are targets of SUMOylation [52–54], whereas parkin and PINK1 are regulated by NEDDylation [13,55]. Here, we demonstrated that parkin is a novel target of ISG15 conjugation. Our current work also proposes that ISG15 modification affects toxic protein accumulation by positively modulating parkin activity. These findings suggest that UBL conjugation is important for maintaining neuronal cell viability, and its alteration could promote neurodegenerative diseases, including PD.

According to previous reports, type I IFNs are key components of proinflammatory pathways via JAK/STAT signalling [56]. Inflammation has been implicated in PD pathophysiology, and inhibition of microglial activation attenuates dopaminergic neuron degeneration in animal models of PD. Studies have shown that inflammatory processes modulate PD risk because higher plasma concentrations of various proinflammatory cytokines correlate with an increased risk of developing PD. Regarding the link between parkin and the inflammatory response, loss of parkin function increases the vulnerability of nigral dopaminergic neurons to inflammation-related degeneration [57]. Moreover, *parkin* mRNA and protein are detectable in brain-resident microglia, astrocytes and peripheral macrophages, and chronic inflammatory conditions reduce parkin levels [58,59]. Specifically, parkin suppresses inflammation and cytokine-induced cell death by promoting proteasomal degradation of E3 ubiquitin-protein ligase TRAF6 [60]. These observations suggest that *parkin* loss-of-function mutations increase susceptibility to inflammation-mediated degeneration of the nigrostriatal pathway and development of PD. This study reveals a novel relationship between inflammation-related factors and parkin. We demonstrate that excessive inflammatory reactions increase the neuroprotective function of parkin through ISGylation, which contributes to the inhibition of nigral dopaminergic neuronal cell death and consequently prevents PD.

We mapped two specific ISGylation sites within parkin: the K349 and K369 residues. Because poly-ISGylation has not been reported, two ISG15 chains are likely to be individually conjugated to these sites. If two ISG15 moieties were conjugated at each site, we would have observed a 1X-ISG-parkin band when cells overexpressed parkin-K349R or parkin-K369R. However, that band was not observed, and both 2X- and 1X-ISG-parkin bands were abolished. These results suggest that a single substitution at either site changes the tertiary structure of parkin, and therefore affects ISG15 conjugation at the other lysine residue.

Native parkin is maintained in an autoinhibited state under resting conditions via intramolecular interactions between the UBL domain and the PUB motif [38]. Here, we showed that ISGylation enhances the ubiquitin E3 ligase activity of parkin. This finding implies that ISGylation might affect parkin autoinhibition and disrupt its inhibitory intramolecular interaction. The activated state of parkin would be triggered and maintained by HERC5. Structural analyses of parkin revealed that the IBR domain is associated with the RING1 domain via a small hydrophobic patch [61]. Moreover, the IBR domain interacts with the RING2 domain over a distance of 50 Å [62]. We demonstrated that the parkin-2KR mutant displays reduced autoubiquitination and thus acts like a dominant-negative mutant. These results suggest that these mutations within the fold of the IBR domain might affect and alter the normal structure of the RING2 domain. This hypothesis is further supported by the finding that intramolecular interaction between parkin^1–80^, which includes the UBL domain, and the parkin^81–465^ fragment, which includes the PUB motif, is inhibited when cells overexpress these two fragments, as well as ISG15-WT, E1 and E2. By contrast, these interactions are enhanced when ISG15-AA is expressed. Therefore, the IBR domain of the parkin-2KR mutant might also interrupt the structural integrity of parkin and binding to the E2 enzyme.

Parkin autoubiquitination is greatly diminished by certain pathogenic missense mutations, including T415N, C418R, G430D, C431F, M434 K and C441R [4,30]. Among these, T415N and C418R are located in the PUB motif, whereas G430D, C431F, M434 K and C441R are located in the RING2 domain. According to X-ray analyses of parkin structure (PDB code-4K95), R33 and R334 residues are located close to the parkin ISGylation sites (K349 and K369), whereas R42 and G328 are located far from these sites [63]. Therefore, mutations R33Q and R334C might interrupt parkin ISGylation through the conformational change in targeting sites and/or domain. Chaugule *et al*. [38] previously showed that the R42P mutant disrupts the fold of the UBL domain, which could inhibit the autoinhibitory interaction of parkin with substrates and consequently maintain parkin in a constitutively active state. Constitutively, active parkin may also have easy access to ISGylation machinery for its modification. Based on these findings, we speculate that inhibition of parkin activity by some of these mutants is largely attributed to their stimulatory effects on autoinhibition. Compared with wild-type parkin, these mutants might maintain the intramolecular interactions and subsequent autoinhibited state more strongly. For conversion of the autoinhibited to the activated state, parkin might require specific effector(s) that relieve the autoinhibitory binding of the UBL domain to the PUB motif.

Taken together, our current findings and the presence of diverse PTMs for parkin regulation highlight the functional importance of parkin within the CNS for the maintenance of cellular homeostasis. In addition, our data support the hypothesis that ISG15 and HERC5 have novel roles that contribute to the pathogenesis of PD.

## Supplementary Material

Supplementary Tables
