# Supplementary material for: Covalent ISG15 conjugation positively regulates the ubiquitin E3 ligase activity of parkin
Source: Open Biol. 2016 Aug 17;6(8):160193. doi: 10.1098/rsob.160193 (PMC5008018; doi:10.1098/rsob.160193)
Supplement: Supplementary Tables [file rsob160193supp1.docx]

**Table S1. List of the primers used to generate parkin constructs encoding wild-type (WT) and its truncation mutants.** The enzyme sites are indicated by italics.

| Name | Vector | Enzyme site | Primers (Forward & Reverse) |
| --- | --- | --- | --- |
| Myc/HA-Parkin WT | pRK5-Myc/HA | MluI  / NotI | 5′-CG*ACGCGT*ATGATGATGTTTGTCA-3′ |
|  |  |  | 5′-AA*GCGGCCGC*CTACACGTCGAACC-3′ |
| Parkin-V5 | pcDNA3.1-V5-His | EcoRI  / XhoI | 5'-CG*GAATTC*ATGATAGTGTTTGTCAGG-3 |
|  |  |  | 5ʹ- AA*CTCGAG*CACGTCGAACCA-3ʹ |
| Myc-  Parkin^81-465^ | pRK5-Myc | MluI  / NotI | 5′-CG*ACGCGT*AATGCAACTGGAGGCGACG-3′ |
|  |  |  | 5′-AA*GCGGCCGC*CTACACGTCGAACC-3′ |
| Myc-  Parkin^226-465^ | pRK5-Myc | MluI  / NotI | 5′-CG*ACGCGT*TTGCACCTGATCGCAACAAA-3′ |
|  |  |  | 5′-AA*GCGGCCGC*CTACACGTCGAACC-3′ |
| Myc-  Parkin^291-465^ | pRK5-Myc | MluI  / NotI | 5′- CG*ACGCGT*GCTGGCTGTCCCAACTCCT-3′ |
|  |  |  | 5′-AA*GCGGCCGC*CTACACGTCGAACC-3′ |
| Myc-  Parkin^381-465^ | pRK5-Myc | MluI  / NotI | 5′-CG*ACGCGT*TTTGAAGCCTCAGGAACAACTACTC-3′ |
|  |  |  | 5′-AA*GCGGCCGC*CTACACGTCGAACC-3′ |
| HA-  Parkin^1-80^ | pRK5-HA | MluI  / NotI | 5′-CG*ACGCGT*ATGATGATGTTTGTCA-3′ |
|  |  |  | 5ʹ- AA*GCGGCCGC*CTACATTTCTTGACCTTTTCTCCACG-3ʹ |

**Table S2. List of the primers used for the site-directed mutagenesis of parkin and HERC5.** The mutation sites are indicated by lines.

| Name | Primers (Forward & Reverse) |
| --- | --- |
| Parkin K299R | 5ʹ-CTGTCCCAACTCCTTGATTAGAGAGCTCCATCACTTCAGG-3ʹ |
|  | 5ʹ-CCTGAAGTGATGGAGCTCTCTAATCAAGGAGTTGGGACAG-3ʹ |
| Parkin K349R | 5ʹ-GAGCCTGACCAGAGGAGAGTCACCTGCGAAGG-3ʹ |
|  | 5ʹ-CCTTCGCAGGTGACTCTCCTCTGGTCAGGCTC-3ʹ |
| Parkin K369R | 5ʹ-CCTTCTGCCGGGAATGTAGAGAAGCGTACCATGAAGG-3ʹ |
|  | 5ʹ-CCTTCATGGTACGCTTCTCTACATTCCCGGCAGAAGG-3ʹ |
| Parkin R33Q | 5ʹ-GAGGTGGTTGCTAAGCAACAGGGGGTTC-3ʹ |
|  | 5ʹ-GAACCCCCTGTTGCTTAGCAAACAACTC-3ʹ |
| Parkin R42P | 5ʹ-GGCTGACCAGTTGCCTGTGATGGGCGCAGG-3ʹ |
|  | 5ʹ-CCTGCGAAAATCACAGGCAACTGGTCAGCC-3ʹ |
| Parkin G328E | 5ʹ-TCCTGCAGATGGAGGGCGTGTTATGCC-3ʹ |
|  | 5ʹ-GGCATAACACGCCCTCCATCTGCAGGA-3ʹ |
| Parkin R334C | 5ʹ-TGTTATGCCCCTGCCCTGGCTGTGGA-3ʹ |
|  | 5ʹ-TCCACAGCCAGGGCAGGGGCATAACA-3ʹ |
| Parkin T415N | 5ʹ-CATCAAGAAAACCAACAAGCCCTGTCCCCG-3ʹ |
|  | 5ʹ-CCGGGACAGGGCTTGTTGGTTTTCTTGATG-3ʹ |
| Parkin C418R | 5ʹ-CCACCAAGCCCCGTCCCCGCTGCC-3ʹ |
|  | 5ʹ-GGCAGCGGGGACGGGGCTTGGTGG-3ʹ |
| HERC5 C994A | 5ʹ-CCCTATAAGAGCACTGACAGCTTTCAGTGTCCTCTTC-3ʹ |
|  | 5ʹ-GAAGAGGACACTGAAAGCTGTCAGTGCTCTTATAGGG-3ʹ |

**Table S3. List of siRNA duplex sequences.**

| Name | Primers (Sense & Antisense) |
| --- | --- |
| HERC5-specific | 5ʹ-GGACUAGACAAUCAGAAAGdTdT-3ʹ |
|  | 5ʹ-CUUUCUGAUUGUCUAGUCCdTdT-3ʹ |
| Control | 5ʹ- CCUACGCCACCAAUUUGGdTdT-3ʹ |
|  | 5ʹ-ACGAAAUUGGUGGCGUAGdTdT-3ʹ |
